# Multiomics analysis of metabolic heterogeneity in cervical cancer cell lines with or without HPV

**DOI:** 10.3389/fonc.2023.1194462

**Published:** 2023-11-23

**Authors:** Xu Liu, Yaqi Zhu, Sheng Huang, Tingyu Shi, Tanghua Li, Yanan Lan, Xiaojian Cao, Yingtao Wu, Jinya Ding, Xiaohua Chen

**Affiliations:** ^1^ Department of Laboratory Medicine, General Hospital of Central Theater Command, Wuhan, Hubei, China; ^2^ School of Basic Medical Sciences, Hubei Minzu University, Enshi, Hubei, China; ^3^ Hubei Provincial Key Laboratory of Occurrence and Intervention of Rheumatic Diseases, Hubei Minzu University, Enshi, Hubei, China; ^4^ Department of Clinical Laboratory, Maternal and Child Health Hospital of Hubei Province, Wuhan, Hubei, China; ^5^ State Key Laboratory of Agricultural Microbiology, College of Veterinary Medicine, Huazhong Agricultural University, Wuhan, Hubei, China; ^6^ The First School of Clinical Medicine, Southern Medical University, Guangzhou, Guangdong, China; ^7^ School of Laboratory Medicine, Hubei University of Chinese Medicine, Wuhan, Hubei, China

**Keywords:** cervical cancer, C33A, CaSki, metabolomics, lipid metabolism

## Abstract

Metabolomics analysis revealed the metabolic heterogeneity of cervical cancer (CC) cell lines C33A and CaSki, and their molecular mechanisms were explored. Using the modified Bligh-Dyer method, the endogenous metabolites of C33A and CaSki cells were divided into polar and nonpolar fractions. The metabolites were analysed by ultra-performance liquid chromatography-quadrupole-time-of-flight mass spectrometry (UPLC-Q-TOF-MS). Then, the differential metabolites were screened by combining multivariate statistical analysis and volcano maps, and functional enrichment and pathway analysis of the differential metabolites were performed. Finally, association analysis was carried out in combination with transcriptomics, and the important differential metabolisms were experimentally verified by real-time PCR (RT−qPCR) and oil red staining. The results showed that between the C33A and CaSki cell lines, there were significant differences in amino acids, nucleotides and lipids, such as in threonine, arachidonic acid and hypoxanthine, in the metabolic pathways. These compounds could be used as markers of differences in cellular metabolism. The heterogeneity of lipid metabolism accounted for 87.8%, among which C33A cells exhibited higher contents of fatty acid polar derivatives, while CaSki cells showed higher contents of free fatty acids and glycerides. Based on correlation analysis of the above metabolic differences in HPV pathways as well as lipid metabolism-related genes, p53 and the genes involved in lipid metabolism pathways, such as Peroxisome Proliferator Activated Receptor Gamma(PPARG) and stearoyl-CoA desaturase (SCD), are relevant to the metabolic heterogeneity of the cells. The differential expression of some genes was validated by RT−qPCR. CaSki cells showed significantly higher glyceride levels than that of C33A cells, as verified by oil red O staining and glyceride assays. The above results showed that the metabolomic differences between C33A and CaSki cells were relatively obvious, especially in lipid metabolism, which might be related to the decreased expression of PPARG and p53 caused by HPV E6. Further studies on the molecular mechanism of lipid metabolism heterogeneity in cervical cancer cell lines with or without HPV could provide a new reference for the development of CC and individualized treatments of tumour patients.

## 1 Introduction

Cervical cancer (CC) is among the three most common gynaecological tumours, and over half a million new cases are diagnosed per year for women worldwide. Persistent high-risk human papilloma virus (HR-HPV) infection is a well-established causative factor in most CCs (1). The pathogenic mechanism of cervical squamous cell carcinoma (CSCC) caused by HPV16 infection, which accounts for 50-60% of cases (2), is mainly due to p53 degradation mediated by the encoded E6 protein (3). However, CC without HPV infection is usually related to the functional mutation of p53 (4). CC primarily metastasizes through lymphatic vessels or direct metastases. Radical hysterectomy, radiotherapy and postoperative cisplatin-based combination chemotherapy are the main therapeutic regimens. However, more than 30% of patients show resistance to radiotherapy, and the recurrence rate of early CC may be greater than 5% within 4.5 years (5, 6). The current standard of radiotherapy for locally advanced cervical cancer (LACC) as well as clinical outcomes has not improved in over 30 years. Multiomics studies have largely broaden our recognition of cancer metabolism. CC induced by oncogenic viruses in carcinogenesis can remain in a precancerous state for several years. Studies aimed at metabolic phenotype of CC will enhance our knowledge on the disturbance of viruses with host cells and the development of CC with or without HPV.

Metabolomics is a field of omics following genomics, transcriptomics, and proteomics that aims to characterize the metabolic profiles of cells, body fluids, or tissues to identify the different metabolites and explore the underlying biological mechanisms (7). The endogenous metabolism of cells is changed during tumour progression. Metabolomics is increasingly being used to discover the key molecular changes behind tumorigenesis; thus, elucidating the various omics differences in model cells is essential. Although transcriptome and proteomic differences in CC cell lines have been reported frequently (8–12), metabolomics has only been reported once. Kalliopi I. Pappa et al. conducted metabolomic analysis using ultra-performance liquid chromatography (UPLC) and high-resolution mass spectrometry (MS) and found that both HeLa and Siha cell lines exhibit Warburg metabolic characteristics and purine salvage pathway activity; in contrast, C33A cells synthesize cytidine through a novel mechanism (13). In this study, we focused on the metabolic differences in polar and nonpolar metabolites of an HPV16-positive CC cell line CaSki established from small intestinal mesenteric metastasis cells and a HPV negative CC cell line C33A. C33A cells tested negative for HPV DNA and RNA. The 273^rd^ codon encoding the p53 protein was changed from arginine to cysteine due to a point mutation. CaSki cells with HPV can lead to p53 degradation. As a recognized tumour suppressor gene, p53 plays a very important regulatory role in the regulation of lipid metabolism in normal cells. The decrease in p53 certainly leads to an imbalance in lipid metabolism in cells (14).

In this study, we adopted UHPLC-quadrupole-time-of-flight mass spectrometry to investigate the metabolomics of CC cell lines between C33A and CaSki to identify the key metabolic changes specific to HPV. We used available transcriptome data to identify the differentially expressed genes that reflect tumour-specific metabolic alterations and further validated them through experiments on corresponding metabolic and genetic changes. Finally, we integrated our metabolic and transcriptome data to reveal the clearly disrupted pathways at the metabolic and transcriptional levels and to identify the potential biomarkers that may contribute to the diagnosis and prognosis of CC, providing a theoretical basis for individualized treatment of patients with clinical tumours.

## 2 Results

### 2.1 Typical total ion counting (TIC) and methodological verification

**Figure 1:**
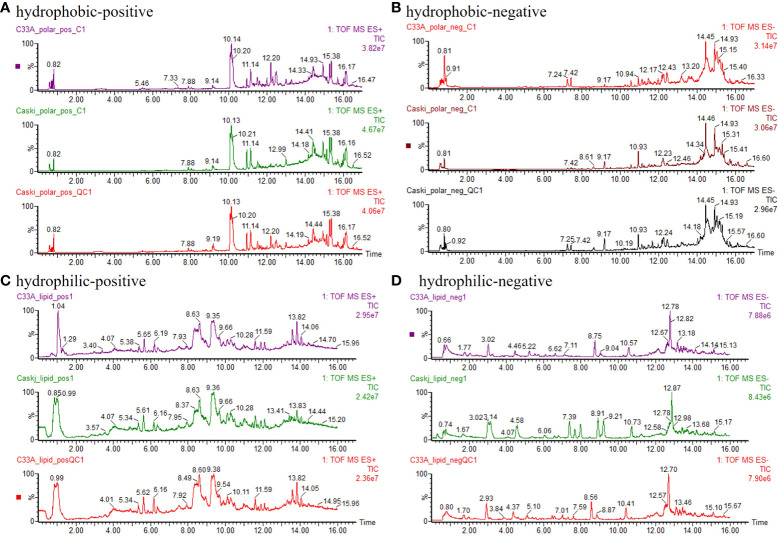
Typical chromatogram. **(A)** hydrophobic components detected by positive ion mode (ESI^+^). **(B)** hydrophobic components detected by negative ion mode (ESI^−^). **(C)** hydrophilic components detected by positive ion mode (ESI^+^). **(D)** hydrophilic components detected by negative ion mode (ESI^−^).

**Figure 1** depicts the TIC of hydrophobic(polar) and hydrophilic(nonpolar) components of the QC group with more chromatographic peaks and better resolution (R) as well as response. The relative standard deviation (RSD) of retention time and peak area for over 85% ions in QC samples were less than 1.0% and 15.0%, respectively, indicating the good stability of the samples and instrument system.

### 2.2 Multivariate statistical analysis

We performed unsupervised principal component analysis (PCA) to verify the quality control (QC) of the metabolic data. The results showed that all C33A, CaSki and QC samples were at 95% Hotelling’s T-squared ellipse and significantly separated into clusters (**Figure S1**) without any outliers in these samples.

**Figure 2:**
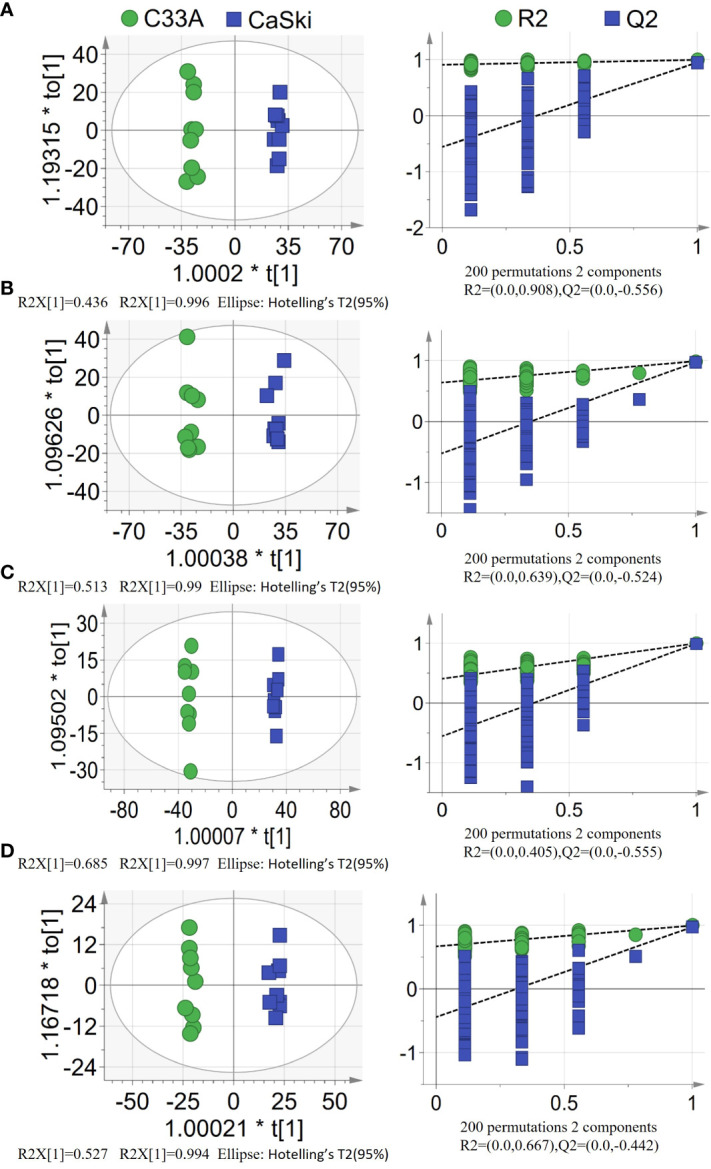
PLS-DA score plots(left) with corresponding permutation test plots (right) derived from **(A)** hydrophobic components detected by positive ion mode (ESI^+^). **(B)** hydrophobic components detected by negative ion mode (ESI^−^). **(C)** hydrophilic components detected by positive ion mode (ESI^+^). **(D)** hydrophilic components detected by negative ion mode (ESI^−^) in LC–MS metabolite profiles between C33A and CaSki.

To obtain a more objective statistical estimation and specific loadings, OPLS-DA for a model discriminating between the samples was conducted based on PCA (**Figure 2**). The OPLS-DA scatter plot of all models (R^2^Y(cum)≥0.99, Q^2^ (cum)≥0.949) presented good R-squared and predictive ability. Partial permutation test results (200 times) showed intercepts of R^2^ ≤0.908 and Q^2^ ≤-0.442 in all the models. If all blue Q^2^ values were lower than the value of the original point or the blue regression line of the Q^2^ point intersected the vertical axis at or below 0 according to the criterion, these PLS-DA models exhibited a lower risk of overfitting. The above results suggested that PLS-DA models could identify the differentially enriched metabolites between the C33A and Caski groups and thus output VIP values of each variable.

### 2.3 Analysis of the metabolite differences between C33A and CaSki

VIP values were generated on the premise of confirming the reliability of the models. Volcano plots were constructed according to the differential fold change of peak area with *p* value of t test (**Figure S2**), metabolites with fold ≥ 1.50 or ≤ 0.67 and *p* value <0.05 were screened, and differential metabolites were determined in combination with VIP ≥ 1.0.

**Figure 3:**
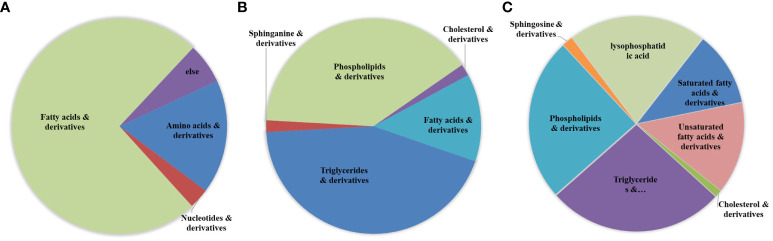
The sector graphs of differential metabolites. Differential metabolites were determined by the use of Lipidview database and Progenesis QI software. Cluster analysis of differential metabolites in the polar group **(A)** as well as the nonpolar group **(B)**. **(C)** Cluster analysis of total lipid metabolites in the metabolome.

Based on information, such as the retention time, precise molecular weight, and secondary fragments, differential metabolites were identified through the Lipidview database and Progenesis QI software. A total of 99 differential metabolites were identified from the polar group, mainly including fatty acid (FA) derivatives, amino acids and lysophospholipids (LPLs). A total of 114 differential metabolites were identified from the nonpolar group, and these metabolites mainly included phospholipids (PL), glycerides and their derivatives, in addition to a small amount of amphiphilic molecules, such as sphingosine derivatives, lysophosphatidic acid (lysoPA), and saturated and unsaturated FAs (**Figure 3**; **Table S1**). Classical univariate ROC curve analysis showed that threonine, arachidonic acid and hypoxanthine could be used as biomarkers to identify C33A and CaSki cells (**Tables S2**, **3**). Among them, metabolites in the polar group with an over 10-fold differences in expression are shown in **Table S2**, and those with an over 10-fold difference in expression in the nonpolar group are shown in **Table S3**.

**Figure 4:**
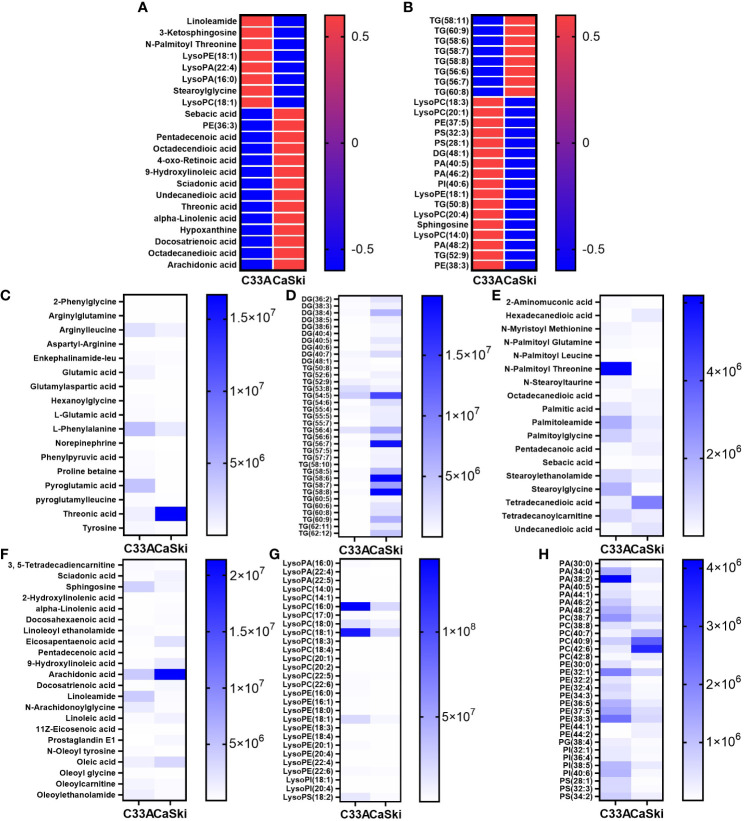
The differential clusters and heat map analysis of all metabolites. The heat map analysis of differential metabolites in the polar group **(A)** and nonpolar group **(B)**. The differential expression analysis of amino acids and derivatives **(C)**, DAG and TAG **(D)**, saturated FAs and derivatives **(E)**, unsaturated FAs and derivatives **(F)**, lysoFAs and derivatives **(G)**, phospholipids and derivatives **(H)**.

MetaboAnalyst 5.0 software was used to analyse the heatmap of polar and nonpolar differential metabolites. Considering the large differences, the intragroup mean values of the top 25 metabolites with the largest differences were selected for heatmap analysis (**Figures 4A, B**). Overall, the levels of LPL, linoleamide, 3-ketosphingosine, N-palmitoyl threonine and stearoylglycine in CaSki cells were significantly lower than those in C33A cells, while FA derivatives with less polar groups, such as sebacic acid, pentadecenoic acid, octadecendioic acid, α-linolenic acid, docosatrienoic acids, octadecanedioic acid, prostaglandin E1, oleic acid, docosahexaenoic acid (DHA) and arachidonic acid, were more highly expressed in CaSki cells than in C33A cells (**Figure 4A**). **Figure 4B** also suggests that CaSki cells had higher triglyceride (TG) contents than C33A cells, while the phospholipid derivatives with polar groups were lower. All the differential metabolites in **Supplementary Table 1** were analysed together. According to their composition characteristics, these differential metabolites could be subdivided into 7 categories, including amino acids, saturated FAs, unsaturated FAs, phospholipids and their derivatives, glycerides, lysoFAs and other metabolites. The differences in the absolute expression of the first six kinds of metabolites are shown in **Figures 4C–H**. Most amino acids, including arginylleucine, L-glutamic acid, L-phenylalanine and phenylpyruvic acid, exhibited higher expression in C33A cells than in CaSki cells. Only threonic acid was less expressed in C33A cells than in CaSki cells (**Figure 4C**). Except for a few TGs with fewer than 54 carbon atoms, such as TG (52:9) and TG (53:8), most diglycerides (DGs) and TGs were more highly expressed in CaSki cells than in C33A cells. The analysis of the correlation between the carbon atom number of triglycerides and cell expression differences showed that TAG with carbon atom numbers between 50 and 53 had higher expression in C33A cells, while those between 54 and 62 showed higher expression in CaSki cells (Spearman correlation coefficient r = -0.676, significance of two-tailed T test *p*=0.000, **Figure S3**). FA derivatives, such as 2-aminomuconic acid, N-myristoyl methionine, N-palmitoyl glutamine, N-palmitoyl threonine, N-stearoyltaurine, palmitoleamide, palmitoylglycine, stearoylethanolamide, stearoylglycine and tetradecanoylcarnitine, were present in high amounts in C33A. Saturated FAs with two carboxyl groups, hexadecanedioic acid, octadecanedioic acid, pentadecanoic acid, tetradecanedioic acid and undecanedioic acid, were at a relatively high level in CaSki (**Figure 4E**). Furthermore, unsaturated FAs that are abundant in C33A cells, such as 3,5-tetradecadiencarnitine, sphingosine, oleoylethanolamide, N-oleoyl tyrosine and linoleoyl ethanolamide, are usually modified by polar compounds. However, those in CaSki cells, such as sciadonic acid, eicosapentaenoic acid, 2-hydroxylinolenic acid, α-linolenic acid, docosahexaenoic acid, 9-hydroxylinoleic acid, arachidonic acid, docosatrienoic acid, oleic acid and prostaglandin E1, were not modified or only hydroxyl groups were added to the unsaturated FA chain (**Figure 4F**). In-depth analysis of 12 kinds of ω-3 and ω-6 FAs and their derivatives showed that only linoleamide (ω-6) and N-arachidonoylglycine (ω-6) had high levels in C33A cells. Compared to CaSki cells, the expression of LPL, which was abundant in C33A cells, was generally not lower; the most significant differences were observed in LysoPA (16:0), LysoPC (18:0), LysoPC (18:1), LysoPE (18:1) and LysoPS (18:2) (**Figure 4G**). Phospholipids and derivatives of PA (38:2), PA (34:0), PE (32:1) and PI (40:6) were highly expressed in C33A cells, while PC (40:7), PC (40:9), PC (42:6), PC (42:8), PE (44:1) and PE (44:2) were highly expressed in CaSki cells (**Figure 4H**). Based on the integrated metabolic data, we found that the metabolic differences between C33A and CaSki cells were mainly focused on lipids. Statistical correlation between the polar distribution of FA metabolites and up/downregulated gene expression in C33A and CaSki cells suggested that most polar and nonpolar FAs (such as triglycerides and diglycerols) had a C33A/CaSki ratio of less than 1. However, most FA derivatives with other polar groups (such as linoleoyl ethanolamide, glycerophosphatidic acid (PA), phosphatidylcholine (PC), phosphatidylglycerol (PG), phosphatidylinositol (PI), phosphatidylserine (PS), phosphatidyl ethanolamine (PE) and LPL derivatives) had a C33A/CaSki ratio of greater than 1. The results above were statistically significant (Spearman correlation coefficient r=0.791, significance of two-tailed T test *p*=0.000, **Figure S4**). That is, C33A cells had a higher content of FA derivatives and stronger polarity than that of CaSki cells. Compared with C33A cells, CaSki cells had a higher content of simple FA chains and weaker polarity.

### 2.4 Enrichment and metabolic pathway analysis of C33A and CaSki

**Figure 5:**
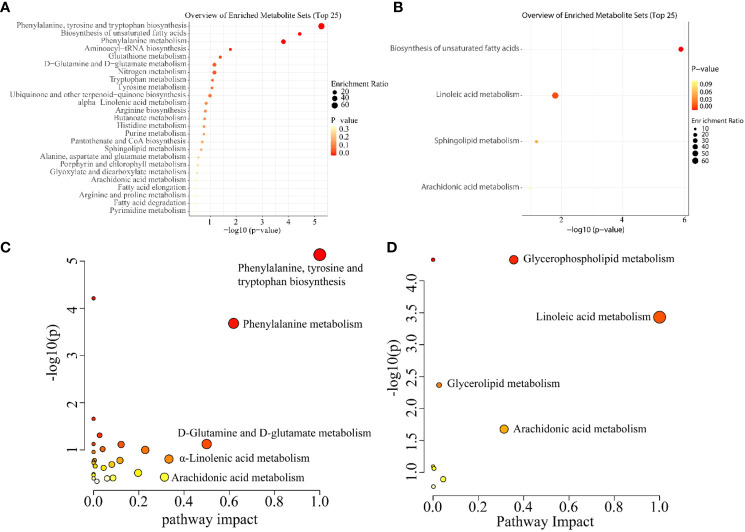
Metabolic enrichment and pathway analysis of C33A and CaSki. Metabolic GO enrichment analysis of enriched metabolite sets (Top25) in the polar group **(A)** and nonpolar group **(B)**. Metabolic pathway analysis of the polar group **(C)** and nonpolar group **(D)**. The horizontal axis represents the pathway impacts and vertical axis represents the -log10(p-value).

Enrichment and metabolic pathway analyses were carried out using MetaboAnalyst5.0 software. The overview of enriched metabolite sets (top 25) is represented by a bubble diagram. The size and colour of the bubble suggest the enrichment ratio and *p* value, respectively (**Figures 5A, B**). The metabolic pathway analysis directly indicated that metabolic pathways with the maximum difference between C33A and CaSki were determined with a pathway impact value ≥0.1 as the standard (**Figures 5C, D**). The results of polar group enrichment analysis showed that C33A and CaSki cells showed significant differences in the biosynthesis of aromatic amino acids (phenylalanine, tyrosine and tryptophan) and unsaturated FAs, as well as phenylalanine metabolism, while linoleic acid metabolism and unsaturated FA biosynthesis were observed in the nonpolar group. Consistent with this conclusion, KEGG analysis also suggested that the differences between C33A and CaSki in the polar group were mainly concentrated on aromatic amino acid biosynthesis (impact=1.0000), phenylalanine metabolism (impact=0.6190), D-glutamine and D-glutamic acid metabolism (impact=0.5000), α-oleic acid metabolism (impact=0.3333) and arachidonic acid metabolism (impact=0.3135), while glycerophospholipid metabolism (impact=0.3562), linoleic acid metabolism (impact=1.0000) and arachidonic acid metabolism (impact=0.3145) were enriched in the nonpolar group.

### 2.5 Transcriptomic analysis and correlation analysis with metabolomics

**Figure 6:**
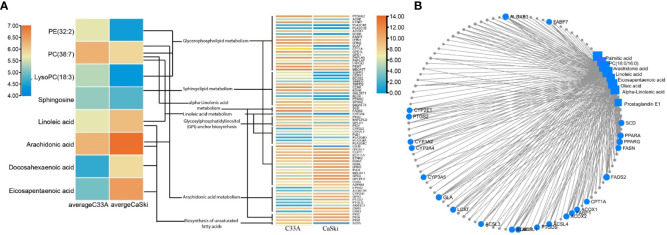
Gene and metabolism correlation analysis. **(A)** Relevant genes associated with different lipid metabolic pathways. **(B)** Correlation analysis of metabolomic and transcriptomic differential genes.

Based on the known transcriptome differences between C33A and CaSki cells in the GEO database, more than 10,000 genes were statistically significant (*p* < 0.05) (**Table S4**). Although the transcriptome pathway analysis could not obviously reveal the lipid metabolism-related pathway, it was observed that the pathway of “human papillomavirus infection” was significantly downregulated in C33A cells compared with CaSki cells (*p*=7.41e^-08^), indicating that the transcriptome data obtained were reliable (**Figure S5**). Through the KEGG and SMPDB websites, we screened P53-related genes and analysed their association with metabolomics. The results showed that P53 pathway-related genes were not directly related to metabolomics. The correlation analysis of HPV-related genes, consistent transcriptomic differential genes associated with metabolomics, showed that only seven HPV pathway genes (*PTGS2*, *P53*, *GNAS*, *BAX*, *TNF*, *VEGFA*, and *PTEN*) were related to lipid synthesis (**Figure S6**; **Table S5**). A total of 391 genes related to metabolic pathways, such as glycerolipid metabolism, unsaturated FA metabolism, linoleic acid metabolism and arachidonic acid metabolism, were screened by the same method, and 244 genes were consistent with transcriptome differences (**Figure 6A**). The 244 genes and 213 differential metabolites were input into the “Network Explorer” module of the MetaboAnalyst5.0 website to analyse the interaction between genes and metabolism in the “Gene−Metabolite Interaction Network” (**Figure 6B**; **Table S6**), demonstrating that there were 95 genes involved in lipid metabolism. The top 10 most relevant genes were FA desaturase 2 (*FADS2*), arachidonate lipoxygenase 3 (*ALOXE3*), peroxisome proliferator activated receptor-γ (*PPARG*), stearoyl-CoA desaturase (*SCD*), peroxisome proliferator activated receptor-α (*PPARA*), galactosidase-α (*GLA*), stearoyl-CoA desaturase 5 (*SCD5*), long chain FA CoA ligase 1 (*ACSL1*), lecithin-cholesterol acyltransferase (*LCAT*) and FA synthase (*FASN*). Except for the unknown functional correlation of *GLA*, the other 9 genes were related to FA metabolism, which further confirmed the difference in lipid metabolism between C33A and CaSki cells.

### 2.6 Lipid metabolism gene validation, oil red assay and total glyceride detection

**Figure 7:**
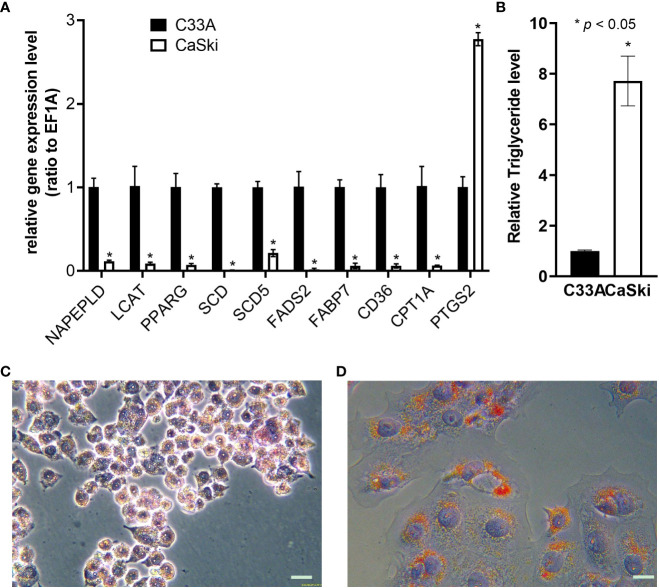
Validation of lipid metabolism of C33A and CaSki. **(A)** Differential expression of the genes related to lipid metabolism through RT-qPCR. **(B)** Detection of differences in triglyceride metabolism. Oil red O staining of C33A **(C)** and CaSki **(D)** cells. *mean p < 0.05.

Real-time fluorescent quantitative PCR primers were designed for the single functional genes of **Table S7** related to metabolism, and the differences in lipid metabolism genes between C33A and CaSki cells were detected (**Figure 7A**). Compared with C33A, the expression of other genes except for prostaglandin peroxide synthase 2 (*PTGS2*) was downregulated in CaSki cells, and the function related to lysoFA synthesis, FA decomposition, desaturation, intake, transportation and metabolism. The results indicated that CaSki contained notably higher TG content than that of C33A (**Figure 7B**). In addition, the oil red assay also confirmed that the lipid content of CaSki was significantly higher than that of C33A (**Figures 7C, D**).

## 3 Discussion

**Figure 8:**
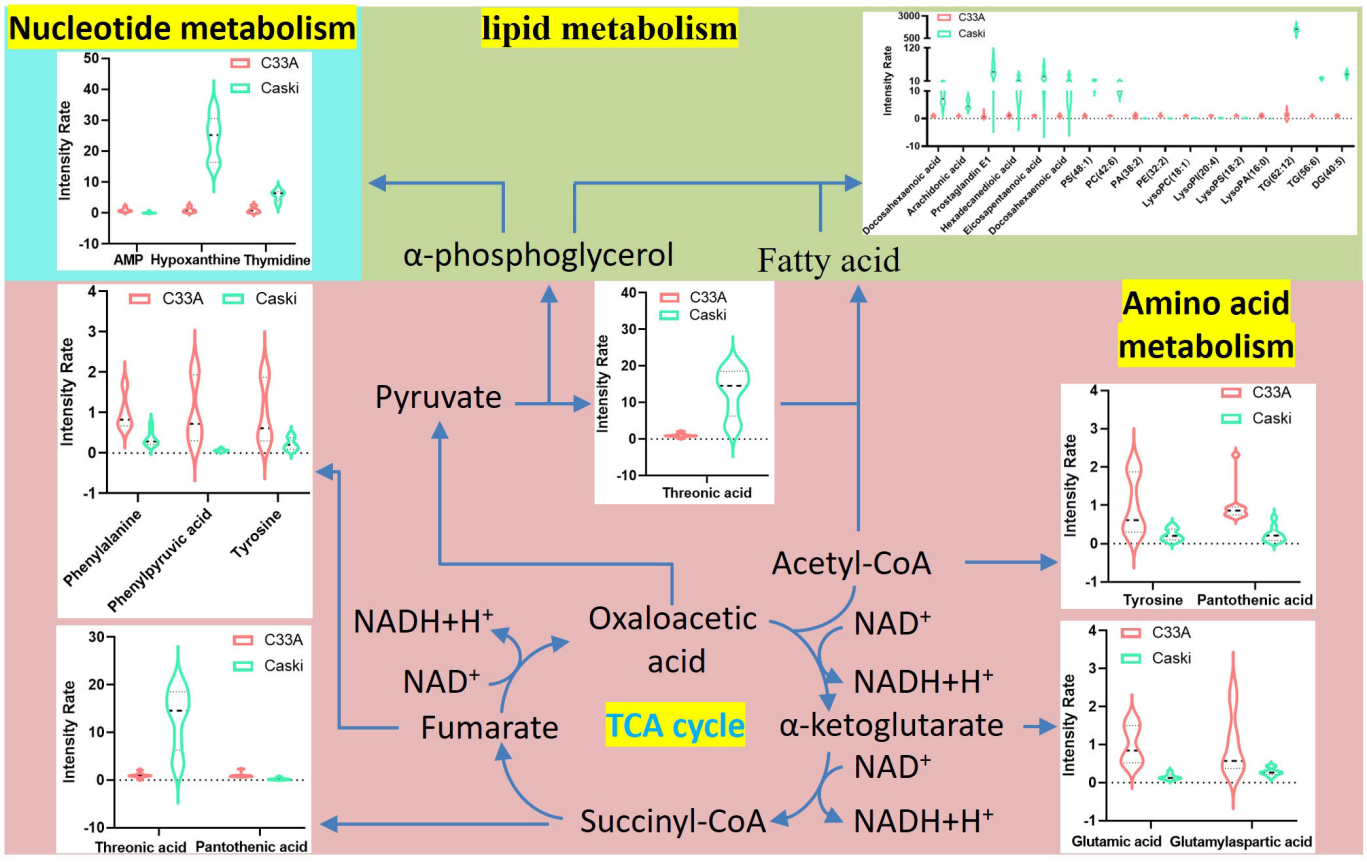
Schematic overview of the correlation analysis of global metabolism in differential metabolites. Violin plots indicate the metabolites detected in our research. The horizontal axis represents different metabolites and vertical axis represents the intensity rate of metabolites.

Metabolomics of tumours is a high-throughput technique used to study the endogenous metabolic changes in the body from the whole and multiple perspectives (7), as well as to clarify the tumorigenesis and development of diseases. Although metabolic biomarkers related to CC have been widely reported, the differences in cell metabolism that distinguish CC with or without HPV have not been studied until now. By analysing the metabolites of C33A and CaSki cells, a total of 99 metabolites were yielded from the positive and negative ion groups of polarity, which were mainly FA derivatives, amino acids and LPLs. A total of 114 different metabolites were confirmed from the positive and negative ion groups of the nonpolarity, mainly phospholipids, glyceride and their derivatives, in addition to a small amount of sphingosine derivatives, lysoFAs, saturated and unsaturated FAs, etc. Regarding amino acid metabolism, the expression of C33A was upregulated in almost all the differential amino acids compared to CaSki, and only threonic acid showed the opposite trend. GO and KEGG analyses indicated that these differences were largely associated with the biosynthesis of phenylalanine, tyrosine, and tryptophan, as well as the metabolism of phenylalanine. Notably, the threonine content in C33A cells was significantly lower than that in CaSki cells (average of C33A/CaSki = 0.0765). However, N-palmitoyl threonine in C33A cells was dramatically higher than that in CaSki cells (average of C33A/CaSki = 431.9820). We speculated that CaSki cells cannot synthesize threonine well into N-palmitoyl threonine, and the function of the latter remains unknown. Threonine participates in lipid metabolism, and several studies have shown that threonine may be a positive regulator of lipid metabolism disorders (15–17). Additionally, the results of polarity group analysis showed that the expression of 4-ketoretinol, hypoxanthine and thymidine was downregulated in C33A compared with CaSki. Conversely, AMP, pantothenic acid, folinic acid and tryptamine were upregulated in C33A compared to CaSki. AMP, hypoxanthine and thymidine are mainly involved in nucleotide metabolism, while 4-ketoretinol, pantothenic acid and tryptamine are primarily involved in lipid metabolism. According to the above metabolic differences, we deduced the overall metabolic differences between C33A cells and CaSki cells, as shown in **Figure 8**. As we know, the TCA cycle is the center of metabolism, and the intermediate products of the cycle are important substrates for the synthesis of amino acids (such as threonine, tyrosine and glutamic acid, etc.). Acetyl-coA produced by the TCA cycle is the most important substrate in fatty acid synthesis. and oxaloacetic acid produces pyruvate through decarboxylation, which can further generate α-phosphoglycerol, which participates in lipid metabolism as a substrate for glycerol ester synthesis, or converted into glucose 6-phosphate, which in turn produces ribose through the pentose phosphate pathway and participates in nucleotide metabolism. Of course, glutamate and other substances in amino acid metabolism are also important substrates for base synthesis. The differences in metabolites and enzyme activity ultimately lead to significant metabolic differences between C33A and Caski cells on the layers of nucleic acid, lipid, and amino acid.

Lipid metabolism changes represented by FA synthesis (FAS) and FA oxidation (FAO) have been increasingly recognized as an important metabolic recombination phenomenon in tumour cells. Tumour cells can also hydrolyse the reserved FAs to maintain cell growth when needed. FAs obtained by FA hydrolysis are decomposed through the mitochondrial FA β-oxidation pathway to produce a large amount of acetyl-CoA to meet the energy demand of rapid cell proliferation. The generated acetyl-CoA can enter the tricarboxylic acid (TCA) cycle or be transported to the cytoplasm as citric acid to synthesize FAs. Excess acetyl-CoA can be esterified with cholesterol or diacylglycerol (DAG) to form cholesterol esters (CE) or triacylglycerol (TG) and be stored as lipid droplets (18). Enrichment pathway analysis of lipids showed that differential metabolites of C33A and CaSki cells were associated with the metabolism of glycerophospholipids, linoleic acid and arachidonic acid. The glycerophospholipids above then decompose one molecule of the FA chain under the exposure of phospholipase to become LPLs. Hydrophobic triglycerides and their derivatives constitute an important component of the cell membrane, and their content accounts for nearly 50% of the total amount of the cell membrane. Different membrane lipid compositions in cells may be a sign of lipid metabolism changes. Our omics analysis demonstrated that phospholipids and derivatives such as PA (38:2), PA (34:0), PE (32:1) and PI (40:6) were highly expressed in C33A cells. PC (40:7), PC (40:9) and PC (42:6) showed upregulated expression in CaSki cells. Correspondingly, LPLs were generally expressed at high levels in both CaSki and C33A cells, and their expression in C33A cells was usually higher than that in CaSki cells, with the most significant differences in LysoPA (16:0), LysoPC (18:0), LysoPC (18:1), LysoPE (18:1) and LysoPS (18:2). LPLs are produced by the hydrolysis of phospholipid-related substances under the catalysis of phospholipase, resulting in the loss of one molecule of FAs. The activity of phospholipase affects its content, but too many LPLs, such as lysophosphatidylcholine (LPC), are harmful to cells and lead to inflammation, oxidative stress damage and cell apoptosis. In cell culture, apoptosis or inflammation caused by high levels of LPCs did not occur in C33A cells. It is possible that other kinds of LPLs disrupt the effect of LPCs.

Another manifestation of lipid metabolic heterogeneity in both CC cell lines (CaSki and C33A) is the difference in the metabolism of unsaturated FAs, which are also known as essential FAs. Linoleic acid and arachidonic acid are unsaturated FAs, which are further divided into monounsaturated and polyunsaturated FAs. Oleic acid and linoleic acid are the most common monounsaturated FAs (MUFAs) and are universally good for health. In contrast, there are four major types of polyunsaturated FAs (PUFAs) (ω-3, ω-6, ω-7 and ω-9). Studies have indicated that ω-6 PUFA metabolites derive inflammatory factors, such as prostaglandin E2 (PGE2) and leukotriene B4 (LTB4), which can stimulate organisms to produce inflammatory cytokines and thus form a microenvironment that promotes tumour growth. The metabolites produced by ω-3 PUFAs are anti-inflammatory factors, such as prostaglandin E3 (PGE3) and leukotriene B5 (LTB5), which inhibit the production of cell inflammation and the growth of tumours. We analysed the differences in the metabolism of ω-3 and ω-6 in C33A and CaSki cells and found no statistical significance in the distribution and content of PUFAs in the two types of cells. However, recent studies have shown that overwhelming PUFA peroxidation is among the main inducers of iron apoptosis (19). Li H et al. studied the metabolomics differences in different cell lines and found that triglycerides (TAG) containing PUFAs can be obviously divided into two clusters. One contained polyunsaturated triglycerides (PUFA^high^) with more unsaturated double bonds (more than 4), and the other comprised monounsaturated triglycerides (MUFA^low^) with fewer unsaturated double bonds. Analysis revealed that the number of unsaturated FAs in triglycerides with metabolic differences in C33A and CaSki cells was not less than 4, but the number of TAG carbon atoms in C33A cells was shorter than that in Caski cells (20).

RT−qPCR analysis of lipid metabolism-related genes in C33A and CaSki cells showed that the expression of *PTGS2* was upregulated in CaSki cells, but the *NAPEPLD, LCAT, PPARG, SCD, SCD5, FADS2, FABP7, CPT1A*, *ACSL1* and *CD36* genes were downregulated compared with C33A cells. *PTGS2* is a key rate-limiting enzyme that catalyses the synthesis of prostaglandin from arachidonic acid. The tendency of mRNA expression corresponded with the metabolomics results. The *NAPEPLD* gene is a kind of phospholipase type D enzyme. The function of *LCAT* is to transfer the unsaturated FA located in C2 of lecithin, which can raise high-density lipoprotein (HDL) levels in the blood to free cholesterol and then generate lysolecithin as well as cholesterol ester. To overcome lipotoxicity, cancer cells overexpress different subtypes of stearoyl-coenzyme A desaturase (SCD). The *SCD1* and *SCD5* genes are both members of the fatty acid desaturase (FADS) family, encoding some proteins involved in FA biosynthesis that catalyse stearic acid and palmitic acid to produce MUFAs (such as oleic acid and palmitoleic acid). When the cells are free of exogenous lipid intake, the inhibition of *SCD1* induces ferroptosis and cell apoptosis (21). However, metabolomics showed that oleic acid, linolenic acid and arachidonic acid levels in CaSki cells were significantly higher than those in C33A cells, possibly because oleoyl glycine, oleoylcarnitine, oleoylethanolamide and N-oleoyl tyrosine were significantly higher in C33A cells than in CaSki cells. Oleic acid in C33A cells was rapidly converted into the above oleic acid derivatives and subsequently responsible for lower oleic acid levels in the cells. *FADS2* is involved in the biosynthesis of PUFAs from essential PUFA precursors, such as linoleic acid and α-linolenic acid. *FABP7* binds to long-chain FAs and other hydrophobic ligands. The role of FABPs includes FA uptake, transportation and metabolism, while *CD36* accelerates FA absorption and transportation. PPARG is a key regulator of adipocyte differentiation and is also closely associated with obesity, diabetes, atherosclerosis and cancer (22). Carnitine palmitoyltransferase 1 (CPT1) converts FA chains into acylcarnitine and shuttles in mitochondria for oxidation and energy production, thereby reducing TG content (23). The results of RT−qPCR also confirmed that *CPT1A* was highly expressed in C33A cells.All the above data indicate abnormal lipid metabolism in Caski cells. However, given the cellular heterogeneity between C33A and Caski, it is difficult to answer the correlation between lipid metabolism abnormalities in Caski cells and the integration of HPV DNA into the genome, Recently, Yang et al (24). reported that Caski cells were subjected to whole genome sequencing using the Nanopore Long Read Sequencing method. The sequencing results showed that there were 448 HPV integrations in the genome of Caski cells, GO analysis of HPV integration site genes showed that this integration led to significant changes in the plasma membrane of Caski cells (**Figure S7**). One of these integrations is right between *GSTM5P1* and *PPARG* (**Table S8**), Coincidently, our RT-qPCR analysis showed that HPV integration led to downregulation of PPARG expression. whether HPV genes drive downregulation of *PPARG* expression deserves further research and clarification. *PPARG* is a key upstream regulatory gene in lipid metabolism (22). The change in its expression may be the root cause of abnormal lipid metabolism in Caski cells. In addition, HPV E6 regulates lipid metabolic pathways in part because of its interaction with p53. p53 is directly related to lipogenesis and carcinogenesis. Unrelated to tumour inhibition, p53 also functions as a novel regulatory factor of liver lipid metabolism through microarray analysis of human hepatogenic cells. Namely, p53 genes regulating lipid metabolism were found to affect systemic lipid homeostasis and the development of atherosclerosis from intracellular ceramide and FA metabolism to the regulation of systemic lipid absorption and lipoprotein metabolism (25). P53 is a kind of lipid regulatory inhibitor that restrains the lipogenesis of sterol-regulatory element binding protein 1c (SREBP1C) (26). In contrast, p53 deficiency promotes lipid accumulation (27). HPV E6 indirectly binds to p53 in host cells through ubiquitination degradation, which damages the normal apoptosis and cell cycle regulation mechanism mediated by p53. Therefore, p53 deficiency in HPV E6-infected cell lines may reconstruct lipid homeostasis in tumour cells. We found that the majority of DG, TG and FAs (such as eicosapentaenoic acid, arachidonic acid, oleic acid, and hexadecanedioic acid) were expressed at a higher level in CaSki than in C33A. This high expression is usually coupled with p53 mutations in HPV-negative CC (4). Approximately 50% of human cancers carry mutant forms of p53 that not only negate the anticancer properties of wild-type p53 but also promote cancer progression (28). In addition, mutated p53 increased the expression of genes involved in FA synthesis (such as fatty acid synthase, FASN). Since the mevalonic acid pathway is associated with malignant characteristics (29–31), its disorder is correlated with mutated p53 and poor prognosis in breast cancer patients. Therefore, we can preliminarily determine that the genotype of CC is associated with poor prognosis by recognizing the differences in lipid metabolites after the interaction between p53 and different types of CC.

We conducted metabolic profile analysis of the CC cell lines C33A and CaSki with or without HPV and first demonstrated significant differences in amino acid, nucleotide and lipid metabolism. Correlation analysis between cellular differential molecules and transcriptomic data showed that transcription differences of lipid genes could commendably reflect lipid metabolism differences in metabolomics in C33A and CaSki. We hypothesized that CaSki cells can reduce the expression of p53 protein, which promotes the synthesis of TG and inhibits lysoFAs as well as polar FA derivatives by regulating lipid metabolism through HPV16 E6. Although there are mutations in the p53 gene of C33A cells, the single site mutation limits the maintenance of p53 in normal FA metabolism. The above conclusions still must be confirmed by further studies. In conclusion, we performed lipid metabolism analysis of CC systems with and without HR-HPV by integrating metabolomics and transcriptomic data, which could facilitate the development of novel therapeutic targets and biomarkers for this disease. Future studies with many patients are necessary to verify these findings and investigate the potential clinical application of this knowledge in CC, in which the high lipid metabolism of CaSki could also provide new directions for the treatment of CC with HR-HPV infection.

## 4 Materials and methods

### 4.1 Instruments and reagents

Acquity™ ultrahigh-performance liquid chromatography (UPLC) system, Xevo G2-XS Q/TOF mass spectrometer (MS), Progenesis QI software and Acquity™ UPLC HSS T3 chromatographic column (100 mm×2.1 mm, 1.8 μm) (Waters, USA); SIMCA software (Umetrics, Sweden); 5427R centrifuge (Ebender AG, Germany); Milli-Q ultrapure water system (Millipore, USA); Chromatography used methanol, acetonitrile, isopropyl alcohol and ammonium acetate (Merck, Germany); FBS, DMEM, RPMI 1640 and penicillin/streptomycin (Gibco, South America); PBS (Biyuntian, China); SYBR GREENI PCR Mix and reverse transcription kit (Vazyme, China); RNA extraction kit (Tiangen, China); Triglyceride detection kit (Elabscience, China); Oil red O staining solution (Solarbio, China).

### 4.2 Cell culture and sample preparation

C33A (HPV negative) and CaSki (HPV16) cervical cell lines were purchased from American Tissue Culture Collection (ATCC). C33A cells were cultured in Dulbecco’s modified Eagle medium (DMEM), and CaSki cells were cultured in Roswell Park Memorial Institute (RPMI) 1640 medium. The medium was supplemented with 10% (v/v) foetal bovine serum (FBS) and 1% penicillin−streptomycin. We cultured the cells at 37°C and 5% CO_2_ for proliferation. Cells were harvested for the assay until they reached a cell density of approximately 1×10^7^ cells/mL. The culture medium was discarded and washed twice with precooled PBS solution. The cells were rapidly quenched in liquid nitrogen, and 600 μL of precooled methanol/water (4:1, v/v) was added to the culture flasks. The cells were carefully scraped out with a cell scraper, suctioned into a 2 mL centrifuge tube and washed again. The cells were finally stored at -80°C for subsequent testing.

The hydrophobic and hydrophilic components of the formulation were separated by the modified Bligh–Dyer method (32), briefly, Cell cultures were concentrated and dried under vacuum, and then 350 μL of precooled methanol/water (2:1, v/v) and methylene chloride were added and mixed for 2 min. Cell suspensions were centrifuged at 4°C and 14000 r/min for 10 min, separating the upper and lower layers. The supernatant was analysed by adding 100 μL acetonitrile/water (2:1, v/v) to the upper layer for dissolution. The lower layer was dissolved in 500 μL of isopropanol/acetonitrile/water (2:1:1, v/v) in a vortex and diluted for analysis.

### 4.3 Chromatography and mass spectrum conditions

Upper layer (polar parts): For metabolite profiling, The mobile phase is 0.1% acetonitrile formate (A) and 0.1% formate water Solution (B), The solvent gradient was as follows: 0.0-3.5 min, 2-3% solvent A; 3.5-7.5 min, 3%-35% solvent A; 7.5-14.5 min, 35%-100% solvent A; 14.5-17.5 min, 100% solvent A. The flow rate was 0.4 mL min^-1^. The chromatographic column temperature was maintained at 40°C.

Lower layer (nonpolar parts): For metabolite profiling, mobile phase A was isopropanol/acetonitrile (9:1, v/v), and mobile phase B was acetonitrile/water (3:2, v/v), with both solvents containing 0.1% methanoic acid and 10 mM ammonium formate. The solvent gradient was as follows: 0.0-2.8 min, 30%-43% A; 2.8-3.3 min, 43%-50% A; 3.3-9.0 min, 50%-70% A; 9.0-14.0 min, 70%-99% A; 14.0-16.0 min, 99% A. The flow rate was 0.35 mL min^-1^. The chromatographic column temperature was maintained at 40°C.

The temperature of electron spray ionization (ESI) we used was 110°C. The capillary voltage was 3.0 kV (-3.0 kV). The cone voltage of the samples was 40 V (-40 V). The desolvation gas (N2) flow was 800 L/h with a temperature of 450°C. The cone gas (N2) flow was 40 L/h, and the scanning range was *m/z* 50-1000. Leucine enkephalin was used for real-time recalibration of the mass axis. The data collection mode is MSe (waters_connect platform).

### 4.4 Data processing and statistics

All MS data, including retention times, *m/z* and ion intensities, were extracted using Progenesis QI software (Waters, Milford, MA, USA), which was applied for noise reduction (NR), peak picking (create markers) by automatic default setting, peak alignment by a reference QC sample with full spectrum and normalization to all components. The matrix was then analysed through principal component analysis (PCA) and orthogonal partial least squares discriminant analysis (OPLS-DA) using SIMCA-P+ software (version 14.0, Umetrics, Sweden). Differential metabolites were identified using the LipidView™ database and Progenesis QI software (Waters, Milford, MA, USA). Volcano plots were constructed by R studio. The raw UPLC-Q-TOF-MS data used in this study have been deposited to the MetaboLights Consortium (https://www.ebi.ac.uk/metabolights/studies) with the dataset identifier MTBLS7515.

The metabolome data were imported into MetaboAnalyst (https://www.metaboanalyst.ca/, Last accessed January 16, 2023). The parameters “normalization by sum”, “log transformation (base 10)” and “auto scaling (mean-centred and divided by the standard deviation of each variable)” were applied for data normalization, logarithmic transformation and standardization scaling. The differences in metabolite expression were analysed in depth, and heatmap analysis and biomarker analysis (classical univariate ROC curve analyses) were conducted according to metabolites. MetaboAnalyst “Network Explorer” was also used for gene and metabolite association analysis.

The transcriptome data of CaSki (GSE158033) and C33A (GSE48926) were downloaded from NCBI GEO DataSets (https://www.ncbi.nlm.nih.gov/). FastQC v0.11.9,TrimGalore v0.6.7 (Babraham Institute, UK) and cutadapt v1.18package (33) were used for data quality control. Additionally, Salmon v1.5.2 (34) was adopted to obtain quantitative gene expression information based on the GRCh38 genome. The data of two groups were normalized using the quantile normalization method. Limma v3.52.4 package (35) was used for differential expression analysis. The differentially expressed genes (| logFC | > 1 and FDR < 0.01) that were subjected to GO and KEGG enrichment analysis through clusterProfiler v4.4.4 (36) were screened according to the threshold value. The bubble chart and histogram were constructed by GraphPad Prism 8.4.3 software(GraphPad Software, USA).

### 4.5 RNA extraction and RT−qPCR detection

RNA was extracted from C33A and CaSki cells without any treatment. The RNA extraction procedure was carried out in strict accordance with the kit instructions. Total RNA was reverse-transcribed using a HiScript III 1st Strand cDNA Synthesis Kit (+gDNA wiper) after concentration determination. The ChamQ Universal SYBR qPCR Master Mix kit was used to detect the expression differences of various FA synthesis genes. EF1A was used as the internal reference gene. The cycle threshold (Ct) value was obtained by RT−qPCR amplification. The relative gene expression difference was calculated by the 2^-ΔΔ^Ct method. The primer sets used in the experiment are listed in **Table S9**).

The PCR system consisted of 10 μL of PCR Mix, 4 μL of template cDNA, 0.5 μL of forwards and reverse primers and 5.5 μL of ddH_2_O. PCR amplification conditions were initial denaturation at 95°C for 30 s, 95°C for 15 s, 60°C for 60 s (fluorescence collection) and 40 cycles in total. The melt curve procedures were 95°C for 30 s, 55°C for 15 s and 95°C for 15 s. The fluorescence was collected every 0.3°C.

### 4.6 Glyceride detection

The cell count was needed before glyceride detection. Approximately 1×10^6^ cells were collected, and 100 μL isopropanol was added into the pellets for the mechanical homogenate, centrifuged 10000 g at 4°C for 10 min, and then 10 μL supernatant was taken for the following experiments. The experiment also included a group of standard and blank wells, which were set in three duplicates, and 250 μL of enzyme working mixture was added to each well and incubated at 37°C for 10 min. The OD values of each well were measured at 510 nm with a microplate reader. The experiment was repeated three times. The GPO-POD method of Elabscience® company was used for the detection of glyceride.

### 4.7 Oil red O staining

The lipid content of the cells was measured using the Oil Red O stain kit (Solarbio, China) in accordance with the corresponding instructions. C33A and CaSki cells were fixed with oil red O stationary liquid for 30 min in this assay until they reached a confluence of 70%-80%. Then, the cells were stained with oil red O staining solution for 15 min, and nuclei were dyed using Mayer haematoxylin for 1-2 min. Oil red O buffer solution was added to distilled water for microscope observation after 1 min.

### 4.8 Statistics and analysis

SPSS 22.0 software(IBM, USA) was used for statistical processing of relevant data. All experiments were independently repeated three times. One-way analysis of variance (ANOVA) was used to compare the means of multiple groups, and the results are presented as the mean ± SD. A *p* value < 0.05 was considered to indicate a statistically significant result. Differential metabolites were screened according to a variable importance in projection (VIP) score of >1.0, multiples of change between groups < 0.5 or > 2.0, and *p* < 0.05 was considered significant. Spearman analysis was used to analyse the correlation between the relative expression level of cells and the difference in metabolites in the two groups. Plots were constructed by Graphpad Prism 8.4.3 (GraphPad Software, USA)in addition to the other software automatically generated images.

## Data availability statement

The datasets presented in this study can be found in online repositories. The names of the repository/repositories and accession number(s) can be found in the article/**Supplementary Material**.

## Ethics statement

Ethical approval was not required for the study involving humans in accordance with the local legislation and institutional requirements. Written informed consent to participate in this study was not required from the participants or the participants’ legal guardians/next of kin in accordance with the national legislation and the institutional requirements.

## Author contributions

Conceived and designed the experiments: XL and XCh. Performed the systematic review: TL, YL and YW. Performed the bioinformatic analyses: YZ and XCa. Performed the experiments: SH and TS. Prepared the manuscript: XL, YZ, JD and XCh. All authors contributed to the article and approved the submitted version.
