# Supplementary material for: Multiomics analysis of metabolic heterogeneity in cervical cancer cell lines with or without HPV
Source: Front Oncol. 2023 Nov 23;13:1194462. doi: 10.3389/fonc.2023.1194462 (PMC10701895; doi:10.3389/fonc.2023.1194462)
Supplement: Supplementary file 1 [file Presentation_1.pptx]

## Slide 1
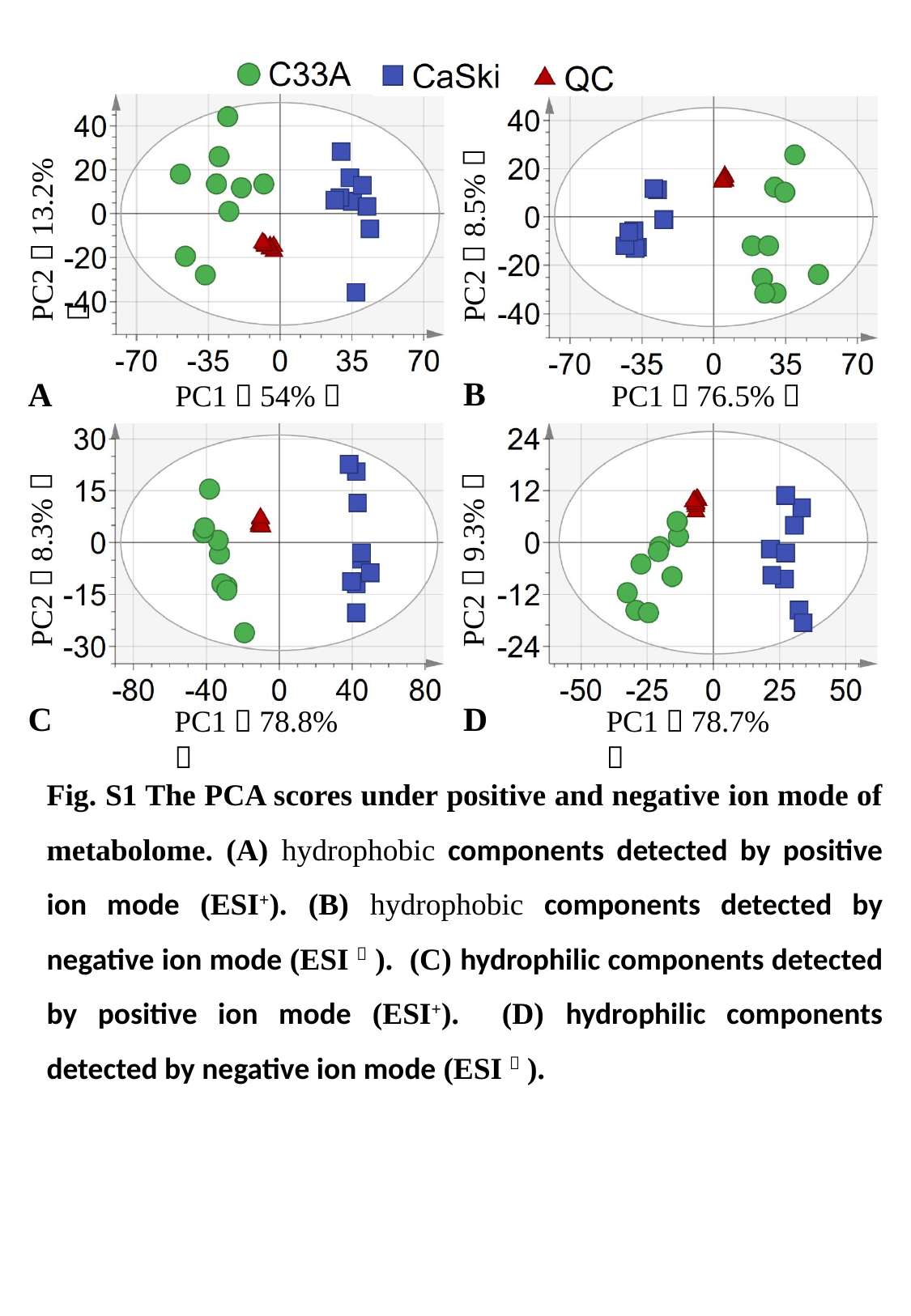

PC2（13.2%）
PC2（8.5%）
B
A
PC1（54%）
PC1（76.5%）
PC2（8.3%）
PC2（9.3%）
D
C
PC1（78.8%）
PC1（78.7%）
Fig. S1 The PCA scores under positive and negative ion mode of metabolome. (A) hydrophobic components detected by positive ion mode (ESI+). (B) hydrophobic components detected by negative ion mode (ESI－). (C) hydrophilic components detected by positive ion mode (ESI+). (D) hydrophilic components detected by negative ion mode (ESI－).

## Slide 2
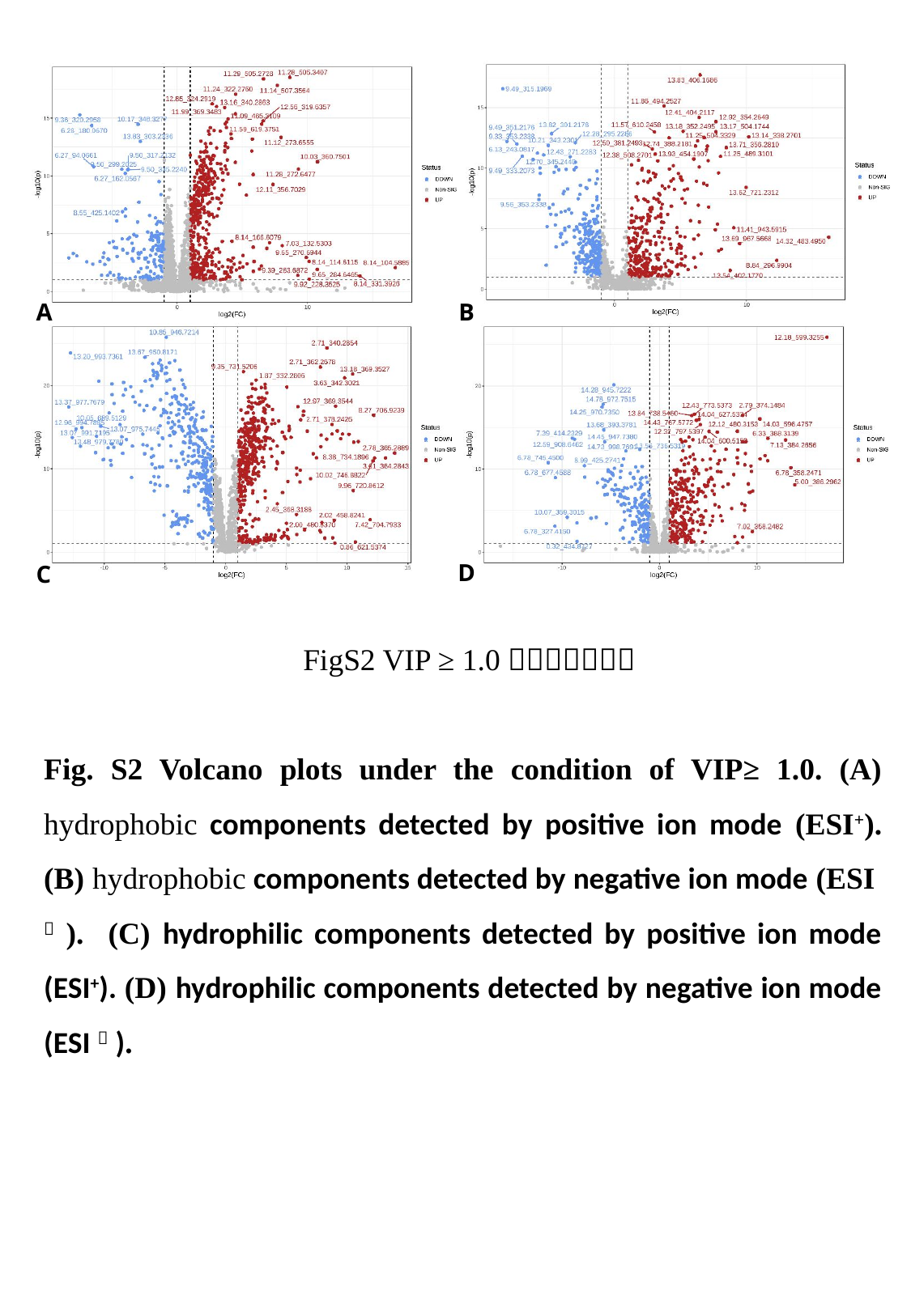

A
B
D
C
FigS2 VIP ≥ 1.0条件下的火山图
Fig. S2 Volcano plots under the condition of VIP≥ 1.0. (A) hydrophobic components detected by positive ion mode (ESI+). (B) hydrophobic components detected by negative ion mode (ESI－). (C) hydrophilic components detected by positive ion mode (ESI+). (D) hydrophilic components detected by negative ion mode (ESI－).

## Slide 3
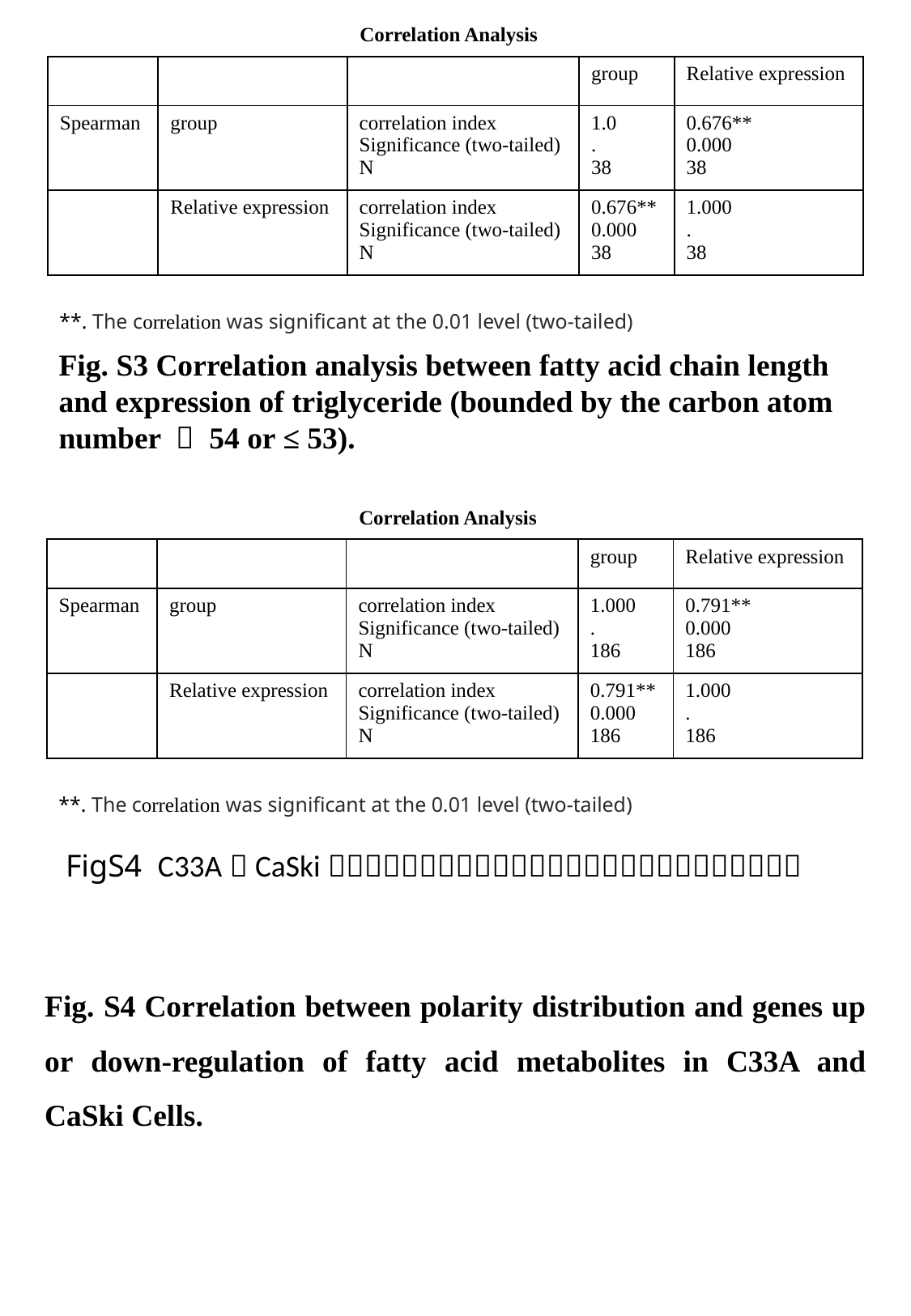

Correlation Analysis
| | | | group | Relative expression |
| --- | --- | --- | --- | --- |
| Spearman | group | correlation index Significance (two-tailed) N | 1.0 . 38 | 0.676\*\* 0.000 38 |
| | Relative expression | correlation index Significance (two-tailed) N | 0.676\*\* 0.000 38 | 1.000 . 38 |
**. The correlation was significant at the 0.01 level (two-tailed)
Fig. S3 Correlation analysis between fatty acid chain length and expression of triglyceride (bounded by the carbon atom number ＞ 54 or ≤ 53).
Correlation Analysis
| | | | group | Relative expression |
| --- | --- | --- | --- | --- |
| Spearman | group | correlation index Significance (two-tailed) N | 1.000 . 186 | 0.791\*\* 0.000 186 |
| | Relative expression | correlation index Significance (two-tailed) N | 0.791\*\* 0.000 186 | 1.000 . 186 |
**. The correlation was significant at the 0.01 level (two-tailed)
FigS4 C33A与CaSki细胞中脂肪酸代谢物的极性分布和基因上下调表达的相关性
Fig. S4 Correlation between polarity distribution and genes up or down-regulation of fatty acid metabolites in C33A and CaSki Cells.

## Slide 4
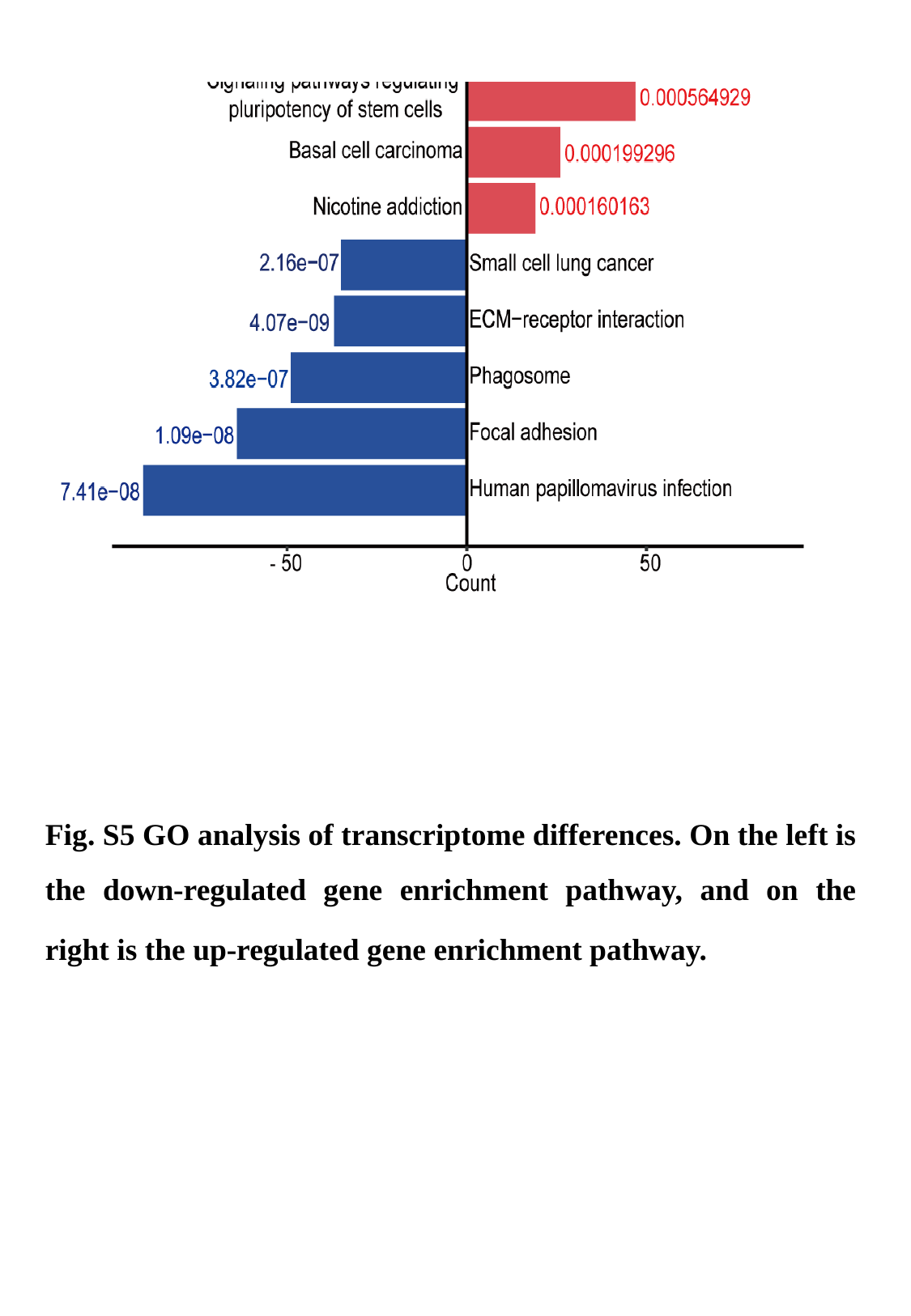

Fig. S5 GO analysis of transcriptome differences. On the left is the down-regulated gene enrichment pathway, and on the right is the up-regulated gene enrichment pathway.

## Slide 5
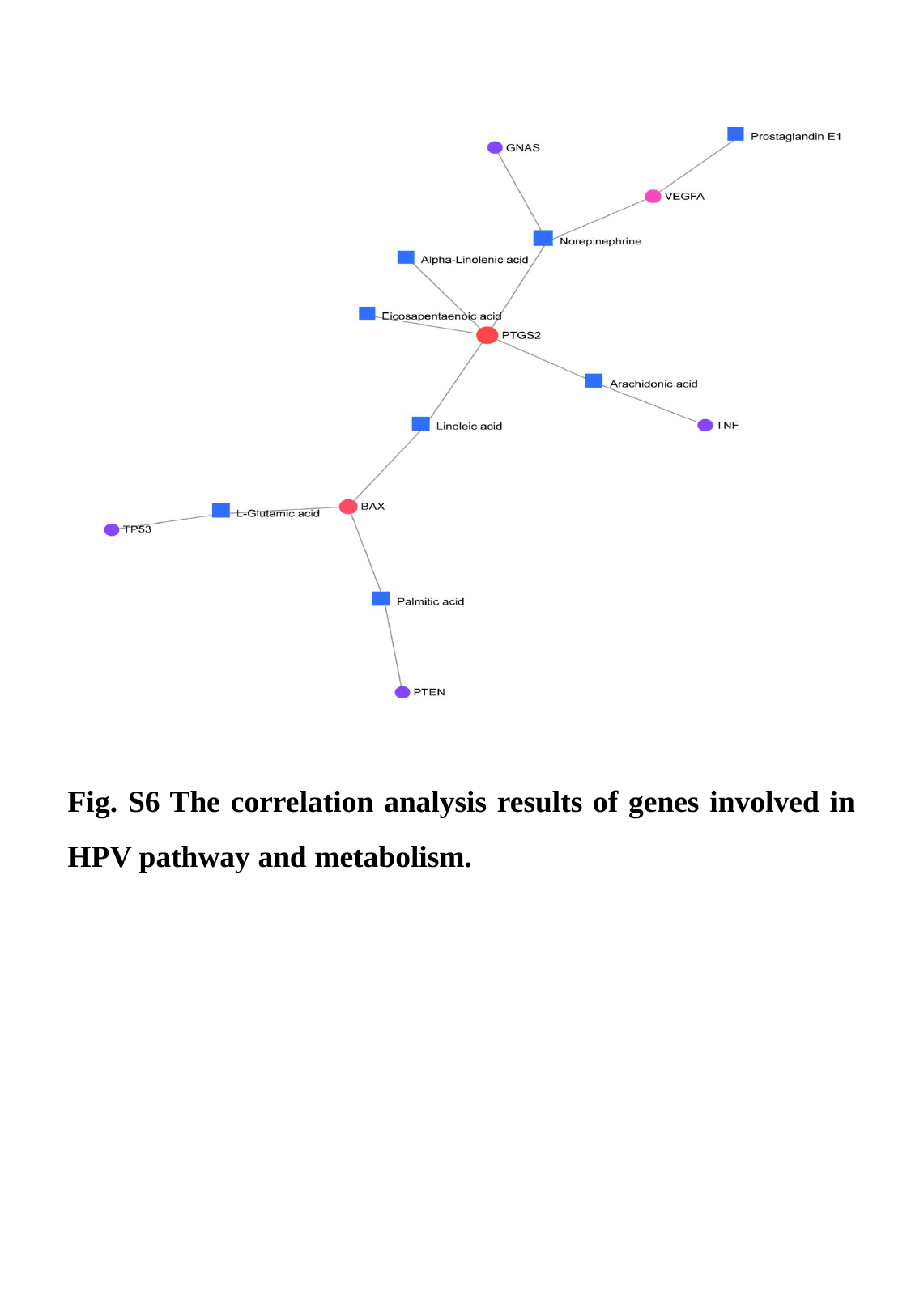

Fig. S6 The correlation analysis results of genes involved in HPV pathway and metabolism.

## Slide 6
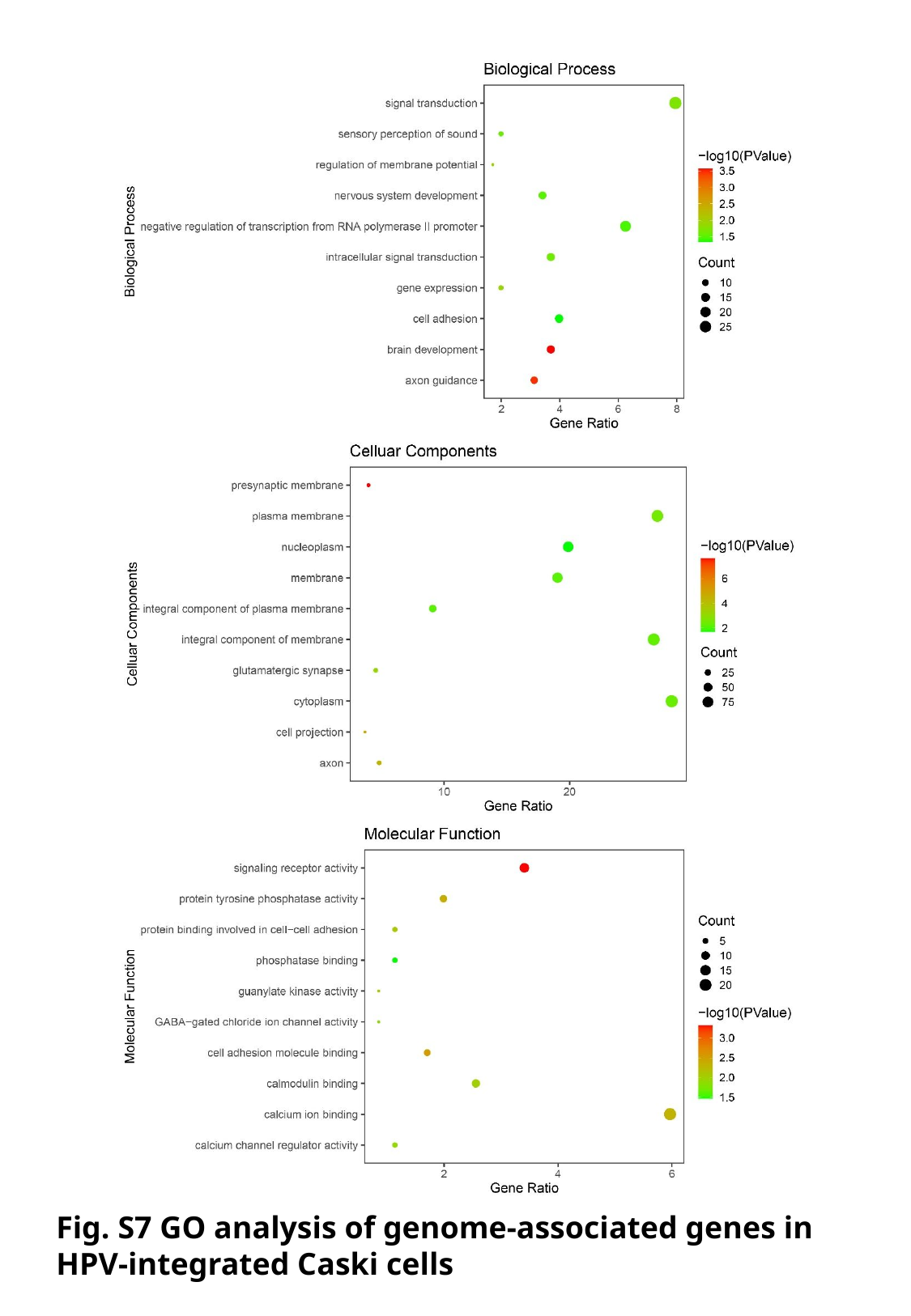

Fig. S7 GO analysis of genome-associated genes in HPV-integrated Caski cells
